# Development and Evaluation of a Human-in-the-Loop Data Curation Training Program to Support a Digital Clinical Trial Platform: Descriptive Feasibility Study

**DOI:** 10.2196/81257

**Published:** 2026-03-25

**Authors:** Mirna Issa, Olivia Yang, Jeffrey Doeve, Shreya Arcot, Amanda Chang, Shriya Amara, Shiven Himanshu Bhakta, Iman Baber, Kawan Shali, Qaiss Dweik, Tony Kin Wai Hung

**Affiliations:** 1 Department of Ecology and Evolutionary Biology, University of California, Los Angeles, Los Angeles, CA, United States; 2 Department of Microbiology, Immunology, and Molecular Genetics, University of California, Los Angeles, Los Angeles, CA, United States; 3 Department of Chemistry and Biochemistry, University of California, Los Angeles, Los Angeles, CA, United States; 4 Department of Neuroscience, University of California, Los Angeles, Los Angeles, CA, United States; 5 Institute for Society and Genetics, University of California, Los Angeles, Los Angeles, CA, United States; 6 Hartford HealthCare Cancer Institute, 85 Retreat Ave, Hartford, CT, 06106, United States, 1 860-972-4183

**Keywords:** clinical trials, undergraduate students, cancer, LookUpTrials, mobile app

## Abstract

**Background:**

LookUpTrials is a clinician-facing digital platform designed to support point-of-care navigation of institution-specific oncology clinical trials, incorporating artificial intelligence–assisted summarization and search functionalities. While embedding curated trial knowledge tools in community oncology workflows is feasible, the human infrastructure required to sustain high-quality, up-to-date trial data over time remains underexplored.

**Objective:**

This study aimed to evaluate the feasibility of a structured training program embedding trainees within a supervised human-in-the-loop workflow for oncology clinical trial data curation in support of the LookUpTrials digital platform.

**Methods:**

We conducted a descriptive feasibility evaluation of a cohort-based training and workflow model. A total of 10 undergraduate trainees curated publicly available trial information from institutional portals and ClinicalTrials.gov across 5 academic medical centers. Trial throughput and participant experiences were summarized descriptively.

**Results:**

Over 10 months, trainees curated 2503 oncology clinical trial entries across 5 institutions, with processing rates increasing over time. Participants reported that structured onboarding and peer support facilitated engagement with data curation workflows.

**Conclusions:**

Standardized human-in-the-loop workflows for clinical trial data stewardship can be implemented with individuals without prior clinical trials or informatics experience when supported by structured training and quality assurance. This study complements prior feasibility work on embedding trial knowledge tools in community oncology settings by focusing on the sustainability of the underlying content pipeline. Further evaluation is needed to assess scalability across institutions and durability over longer periods of platform maturation.

## Introduction

Access to timely, accurate, and institution-specific information about available oncology clinical trials remains a persistent operational challenge in routine cancer care [1-5]. Trial information is often fragmented across registries, institutional portals, and protocol documents, limiting clinicians’ ability to efficiently surface relevant studies during time-constrained visits [2,3,5,6]. Digital clinical trial platforms have emerged to aggregate and present structured trial information at the point of care, but their real-world effectiveness depends not only on technical design and usability but also on the availability of high-quality, up-to-date trial data [7-12].

LookUpTrials is a clinician-facing digital platform designed to surface institution-specific oncology clinical trial information at the point of care. The platform integrates artificial intelligence (AI)–assisted protocol summarization and search functionalities to facilitate trial navigation in routine clinical workflows. Prior work has demonstrated the feasibility of embedding LookUpTrials within community oncology workflows [13,14]. However, sustaining such platforms requires ongoing data stewardship to ensure accuracy, completeness, and local operational relevance, including site-specific trial availability, investigator contacts, and recruitment status [2,15,16]. Scalable workforce models to support this data stewardship layer remain underexplored [2,16].

Human-in-the-loop approaches are increasingly recognized as essential complements to automated pipelines and registry-based feeds, which often lack the granularity and local context required for operational trial navigation [17-19]. In this study, undergraduate trainees were selected as a pragmatic test case to evaluate whether individuals without formal clinical or informatics training can be onboarded into standardized data curation workflows with appropriate supervision and quality assurance. This cohort provides a controlled environment to prototype training materials, workflow design, and quality control processes that could be adapted to more durable staffing models, such as research coordinators, centralized trial operations teams, or hybrid human-AI review pipelines.

We therefore evaluated the feasibility of a structured undergraduate training program embedding trainees within a supervised human-in-the-loop workflow for oncology clinical trial data curation supporting LookUpTrials. This work complements prior feasibility studies of embedding trial knowledge tools in community oncology by focusing on the sustainability of the underlying content pipeline rather than downstream clinical adoption or enrollment outcomes [20,21].

## Methods

### Study Design and Setting

**Figure 1.**
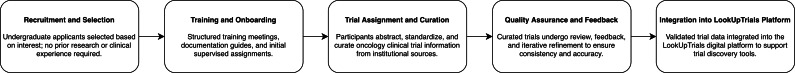
Overview of the undergraduate training and data curation workflow supporting the LookUpTrials platform. The figure illustrates the sequential processes through which undergraduate participants are recruited, trained, and engaged in oncology clinical trial data curation, including data abstraction, peer-led quality assurance, faculty review, and integration into the LookUpTrials platform.

We conducted a feasibility evaluation of a structured training program embedding trainees within a supervised human-in-the-loop workflow for oncology clinical trial data curation in support of the LookUpTrials digital platform (Figure 1). The primary objective was to assess whether individuals without prior clinical trials or informatics experience could be onboarded into standardized data stewardship workflows with appropriate supervision and quality assurance. The evaluation focused on workflow feasibility and participant experience rather than clinical adoption or patient-level outcomes.

### Participants

A total of 10 undergraduate trainees from a university-based student organization were recruited through a competitive application process based on interest in clinical research, availability for longitudinal participation, and commitment to a defined weekly time allocation. Participants had heterogeneous academic backgrounds and varying levels of prior research experience, reflecting the study’s intent to assess the trainability of standardized workflows among individuals without formal clinical or informatics training.

### Training and Workflow Design

Trainees underwent structured onboarding that included orientation to oncology clinical trial concepts, navigation of public trial registries and institutional trial portals, and use of standardized data abstraction templates. Training materials included written documentation, example entries, and step-by-step workflow guides. Trainees were embedded within a supervised, multitiered human-in-the-loop workflow that incorporated peer review and faculty oversight to ensure data accuracy, consistency, and completeness. Iterative updates to training materials and workflow documentation were made based on trainee feedback and observed sources of error.

### Data Sources and Curation Process

Trainees curated publicly available oncology clinical trial information from institutional clinical trial portals and ClinicalTrials.gov across 5 academic medical centers. The sampling frame included all oncology clinical trials listed as active or recruiting on participating institutions’ public trial portals during the study period. Trials were selected systematically based on portal availability and recruitment status rather than through random sampling. Data collection was limited to 5 academic medical centers selected based on the availability of public institutional trial listings and existing program partnerships. The number of trials curated per institution was not intentionally balanced and varied according to institutional trial volume and portal structure.

Institutional clinical trial portals were used to capture site-specific information, such as local recruiting status and institution-level trial availability, which is not consistently available or timely in ClinicalTrials.gov. These data are necessary to support the institution-specific trial discovery functionality of the LookUpTrials platform. Extracted data elements included trial identifiers, disease site, eligibility descriptors, phase, recruiting status, and site-specific information. Data were entered into standardized templates designed to support downstream integration into the LookUpTrials platform. Quality assurance included peer review of entries and secondary verification by program leads to resolve discrepancies and clarify ambiguous protocol information.

### Outcomes and Measures

The primary feasibility outcomes included trial throughput (number of trials curated over time) and descriptive changes in individual processing rates across the study period. The secondary outcomes included participant-reported experiences with onboarding, workflow clarity, peer support, and perceived learning, assessed via postprogram surveys with Likert-scale and open-ended items. Given the exploratory nature of the study and small sample size, analyses were descriptive and focused on characterizing workflow performance and participant engagement rather than formal hypothesis testing.

### Ethical Considerations

This study was conducted as an educational program evaluation and did not meet the definition of human subjects research. The project evaluated a training workflow and analyzed program-level feasibility outcomes using aggregated data and deidentified participant feedback. No identifiable private information was collected, and no patient-level data or protected health information were accessed. All clinical trial information was obtained from publicly available registries and institutional trial portals.

In accordance with US Department of Health and Human Services regulations for the protection of human subjects (45 Code of Federal Regulations 46), this work did not require institutional review board review or approval [22]. As the study did not involve human subjects research as defined by these regulations, informed consent was not required. Participant responses were collected voluntarily and analyzed in aggregate, and no financial compensation was provided.

## Results

### Participant Characteristics

Participant demographic characteristics are summarized in Table 1. In total, 10 undergraduate trainees participated in the program across the study period. Participants represented diverse academic majors within the life sciences and had heterogeneous prior research experience. Several participants reported no prior exposure to oncology or clinical trials at baseline. This heterogeneity aligned with the study objective of evaluating the feasibility of onboarding individuals without formal clinical or informatics training into standardized data curation workflows.

**Table 1.** Demographic characteristics of undergraduate trainees participating in the clinical trial data curation workflow (n=10).

Characteristics	Values, n (%)
Sex	
Male	4 (40)
Female	6 (60)
Academic standing	
Sophomore	4 (40)
Junior	6 (60)
Academic major	
Biology	3 (30)
Biochemistry	2 (20)
Human Biology and Society	1 (10)
Microbiology, Immunology, and Molecular Genetics	3 (30)
Neuroscience	1 (10)
Cohort	
Cohort 1	6 (60)
Cohort 2	4 (40)
Prior research experience (years)	
None	5 (50)
<1	2 (20)
1‐2	2 (20)
>2	1 (10)

### Trial Curation Output and Coverage

The number of curated oncology trials by institution is summarized in Table 2. Over a 10-month period, trainees curated a total of 2503 oncology clinical trial entries sourced from publicly available institutional trial portals and ClinicalTrials.gov across 5 academic medical centers. The volume of curated trials varied by institution, reflecting differences in the size and scope of publicly listed oncology trial portfolios. Curated trials spanned multiple disease sites and trial phases, providing broad coverage of oncology clinical research activity represented within the source portals (Table 3).

**Table 2.** Processed oncology clinical trials by institution across 5 academic medical centers (n=2503).

Institutions	Processed trials, n (%)
UCLA^a^	311 (12.4)
Cedars-Sinai Medical Center	92 (3.7)
University of California, Irvine	584 (23.3)
University of California, San Francisco	1004 (40.1)
University of California, San Diego	512 (20.5)

a UCLA: University of California, Los Angeles.

**Table 3.** Processed oncology clinical trial entries by cancer type across all participating institutions (n=1790).^a^

Cancer types	Processed trials, n (%)
Head and neck	85 (4.7)
Gastrointestinal	211 (11.8)
Gynecologic	85 (4.7)
Breast	113 (6.3)
Sarcoma	73 (4.1)
Lung	156 (8.7)
Central nervous system	155 (8.7)
Skin	175 (9.8)
Genitourinary	178 (9.9)
Hematologic	267 (14.9)
Miscellaneous	292 (16.3)

a Trials classified under nonspecific disease categories (eg, “solid tumors”) were not assigned to a single cancer type and therefore were not included in this distribution.

### Workflow Performance Over Time

**Figure 2.**
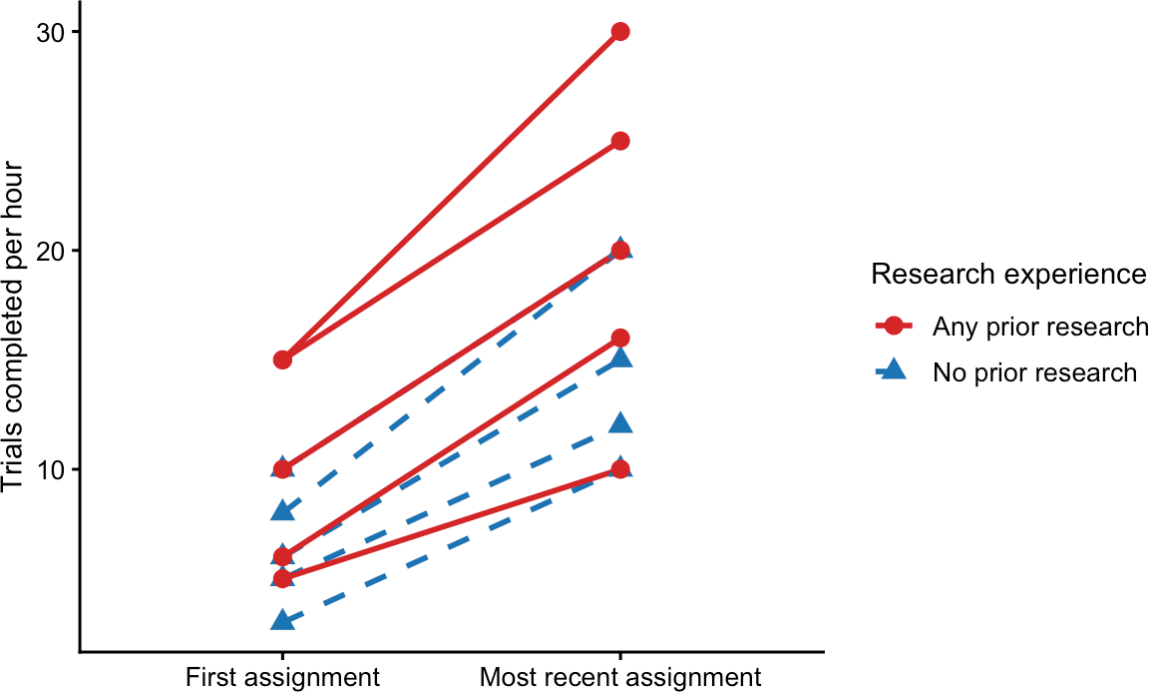
Individual changes in trial processing rates over time (n=10). Each line represents a single participant, illustrating trial processing rates measured during their first assigned trial abstraction and their most recent completed trial abstraction within the study period. Line style and symbol indicate prior research experience (dashed lines with triangles=no prior research experience; solid lines with circles=any prior research experience). The trial processing rate was defined as the number of oncology clinical trials fully abstracted and standardized per hour. Data are presented descriptively to illustrate within-participant variation.

A descriptive analysis of individual trial processing rates indicated increases in throughput over time for most trainees (Figure 2). These changes were consistent with increasing familiarity with trial structures, data abstraction conventions, and workflow expectations rather than formal performance benchmarking. Variability in processing rates across trainees was observed, reflecting differences in baseline experience, availability, and learning trajectories.

### Quality Assurance and Workflow Iteration

Throughout the study period, peer review and secondary verification by program leads identified common sources of ambiguity in trial protocols, including inconsistent terminology across institutional portals and variability in how eligibility criteria and recruitment status were reported. Iterative refinements to training materials and workflow documentation were made in response to these challenges. Over time, fewer clarifications were required per curated trial, suggesting increasing alignment with standardized abstraction conventions and quality assurance processes.

### Participant-Reported Experience

**Figure 3.**
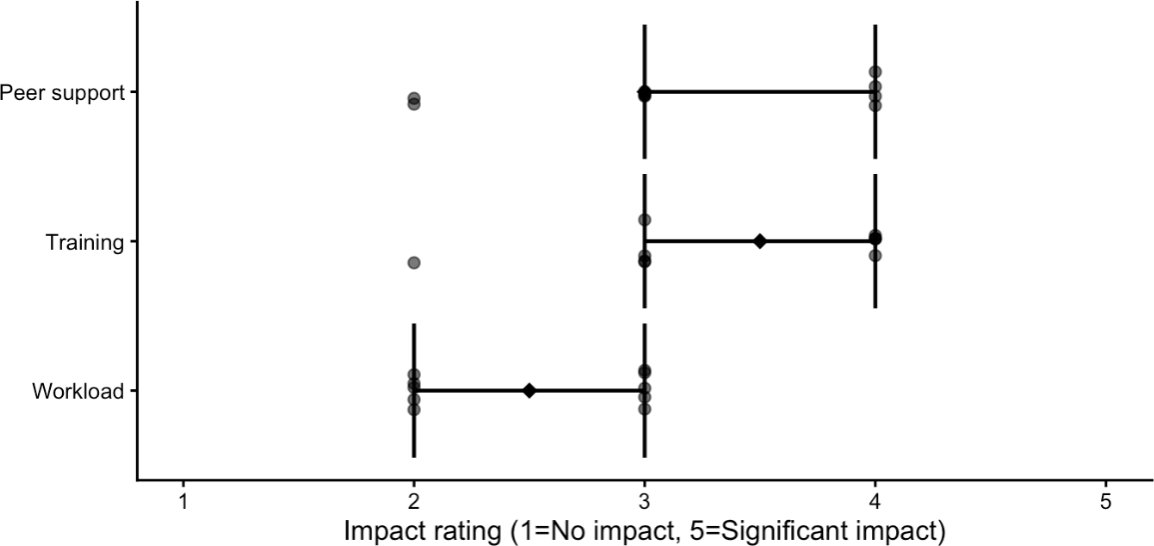
Participant ratings of selected program components (n=10). Points represent individual participant ratings on a 5-point Likert scale. Diamonds indicate median values, and horizontal bars represent the IQR. Components correspond to modifiable elements of the undergraduate training and data curation workflow (eg, training resources, peer support, and workload) and are presented descriptively to illustrate variability in participant perceptions.

Postprogram surveys indicated that participants perceived structured onboarding, shared documentation, and peer support as helpful components of the workflow. Participant ratings of select program components are shown in Figure 3. Trainees reported increased familiarity with clinical trial structure, eligibility terminology, and the role of structured trial information in supporting digital clinical research tools. Participants also noted challenges related to interpreting heterogeneous trial descriptions across institutions and managing time commitments alongside academic responsibilities (Textbox 1). Qualitative feedback suggested that the supervised, iterative workflow design supported learning and engagement despite these challenges.

Textbox 1. **Principal challenges and solutions encountered by participants.**

**Challenges**

- Lack of uniformity between clinical trial sites and dynamic statuses of clinical trial progress
- Difficulty in the production of accurate and cohesive data compilations among multiple program members
- Lack of research experience among program members and background knowledge on oncology broadly
- Ensuring consistent satisfaction and adequate use of time among program members

**Solutions**

- Emphasis on quality control and consistent collaborations to discuss arising issues and uncertainties among program members
- Use of standardized training materials, shared data abstraction templates, and hands-on onboarding to promote consistency
- Use of a shared communication platform and open access to members’ previous work for review
- Open dialogue among program members and communicating feasible deadlines to support sustained participation

## Discussion

In this feasibility evaluation, we found that a standardized, supervised human-in-the-loop workflow for oncology clinical trial data curation can be implemented with individuals without prior clinical trials or informatics training. Over a 10-month period, trainees curated more than 2500 trial entries across 5 academic medical centers, with descriptive increases in processing rates over time and favorable participant-reported experiences with structured onboarding and peer support. These findings suggest that with appropriate workflow design, training materials, and quality assurance processes, nonclinician trainees can contribute meaningfully to the data stewardship layer required to support real-world digital clinical trial platforms.

Digital clinical trial platforms increasingly integrate automated data pipelines and AI-assisted features to support point-of-care trial navigation. However, our findings reinforce that human-in-the-loop data stewardship remains a critical complement to automation, particularly for maintaining institution-specific trial information that is operationally relevant but inconsistently represented in registries. The observed learning curves and workflow stabilization over time highlight the importance of standardized abstraction conventions, shared documentation, and multitiered quality assurance in sustaining high-quality trial data. From an informatics perspective, this work underscores that the success of AI-enabled clinical research tools depends not only on algorithmic performance but also on the design of scalable sociotechnical workflows that maintain the underlying data infrastructure.

This study complements prior feasibility evaluations of embedding LookUpTrials within community oncology workflows by focusing on the sustainability of the content pipeline rather than downstream clinical adoption or enrollment outcomes [13,14]. Together, these findings support a layered model of digital clinical trial platforms in which clinician-facing tools can be embedded within routine workflows, and the underlying data stewardship processes required to maintain those tools can be supported through structured human-in-the-loop workflows. Framing digital trial platforms as sociotechnical systems with interdependent technical and human components may inform more durable implementation strategies as such tools scale across institutions.

Several limitations warrant consideration. First, the sample size was small and drawn from a single undergraduate program, which may limit generalizability to other trainee populations or institutional contexts. Second, while peer review and secondary verification were incorporated into the workflow, formal benchmarking of data accuracy, interrater reliability, or error rates against gold-standard sources was not performed in this feasibility evaluation. Third, the evaluation focused on workflow feasibility and participant experience rather than clinical outcomes, platform usability, or downstream effects on trial referral or enrollment. Finally, this study reflects an early-stage implementation context, and workflow performance may differ in more mature operational settings or under sustained production demands.

Future work should evaluate the scalability and durability of standardized human-in-the-loop workflows across diverse workforce models, including funded research coordinators, centralized trial operations teams, and hybrid human-AI review pipelines. Incorporating formal data quality metrics, automation-assisted abstraction, and continuous quality monitoring may further strengthen the sustainability of trial data stewardship processes. As digital clinical trial platforms mature, integrating these workflow designs into enterprise research operations could help ensure that AI-enabled tools remain supported by reliable, up-to-date, and operationally relevant trial data over time.

## Abbreviations

AI: artificial intelligence
